# Seborrheic Pemphigus: A Misunderstood Variant of Pemphigus Foliaceus

**DOI:** 10.7759/cureus.59389

**Published:** 2024-04-30

**Authors:** Diana Gallegos Espadas, Arely Gissell Ramirez Cibrian, Jesús Iván Martínez-Ortega

**Affiliations:** 1 Internal Medicine, Hospital Regional Elvia Carrillo Puerto, Mérida, MEX; 2 Medical Benefits, Mexican Institute of Social Security, Campeche, MEX; 3 Dermatología, Instituto Dermatológico de Jalisco, Zapopan, MEX

**Keywords:** acantholysis, seborrheic pemphigus, pemphigus foliaceus, desmoglein 1, pemphigus erythematosus

## Abstract

Seborrheic pemphigus (SP) represents a localized and superficial form of pemphigus foliaceus (PF) often mistaken for other dermatological conditions such as seborrheic dermatitis (SD) due to clinical similarities. Additionally, SP may be conceptually confused with pemphigus erythematosus (PE) due to historical terminology and overlapping clinical features. We present a case study of a 38-year-old female initially diagnosed with SD but later identified as SP through detailed clinical and histopathological analysis. We discuss the challenges in accurately diagnosing SP, emphasizing the importance of distinguishing it from PE and other acantholytic dermatoses. Furthermore, we highlight the effectiveness of topical treatment in managing SP, contrary to the systemic therapy often required for PE. Our findings underscore the necessity for further research to optimize management strategies for SP and emphasize the significance of precise terminology in clinical practice and research.

## Introduction

The term "pemphigus" derives from the Greek "pem-phys," meaning blister or bubble [1]. Although it's a rare condition, it's the most prevalent within the group of immunobullous diseases. Two primary variations of pemphigus exist: pemphigus vulgaris and pemphigus foliaceus (PF) [1].

PF is a persistent autoimmune blistering condition affecting the skin. It's marked by the presence of autoantibodies targeting an element of the epidermal desmosome called desmoglein 1 (Dsg1). When these antibodies bind to the antigen, it triggers the formation of splits beneath the upper layer of the skin, leading to the detachment of keratinocytes and a loss of cellular adhesion (acantholysis) primarily in the superficial epidermis and clinically to the development of flaccid bullae [1,2].

We present a localized form of PF resembling seborrheic dermatitis and conduct a detailed analysis of its differential diagnosis, classification, and treatment.

## Case presentation

**Figure 1:**
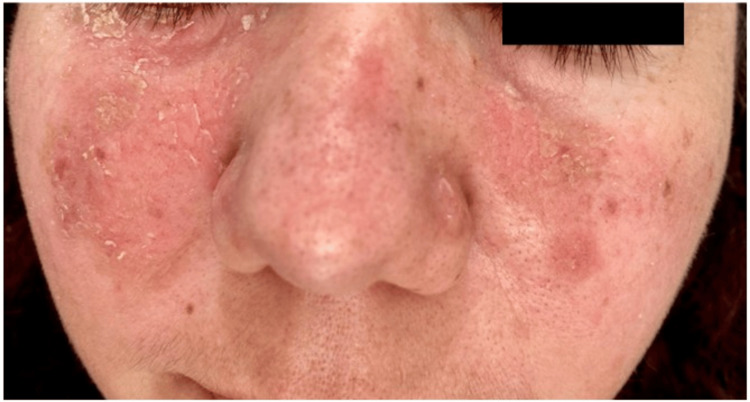
Clinical presentation showing erythematous scaly plaques forming a butterfly-shaped pattern on the nose and cheeks

A 38-year-old woman with no notable family history, drug usage, or chronic illnesses presented with a butterfly-shaped rash on her cheeks, persisting for one year and accompanied by itching, previously treated as seborrheic dermatitis for seven months without improvement. Physical examination revealed erythematous scaly plaques on the nose and cheeks in a butterfly-shaped pattern (Figure 1).

**Figure 2:**
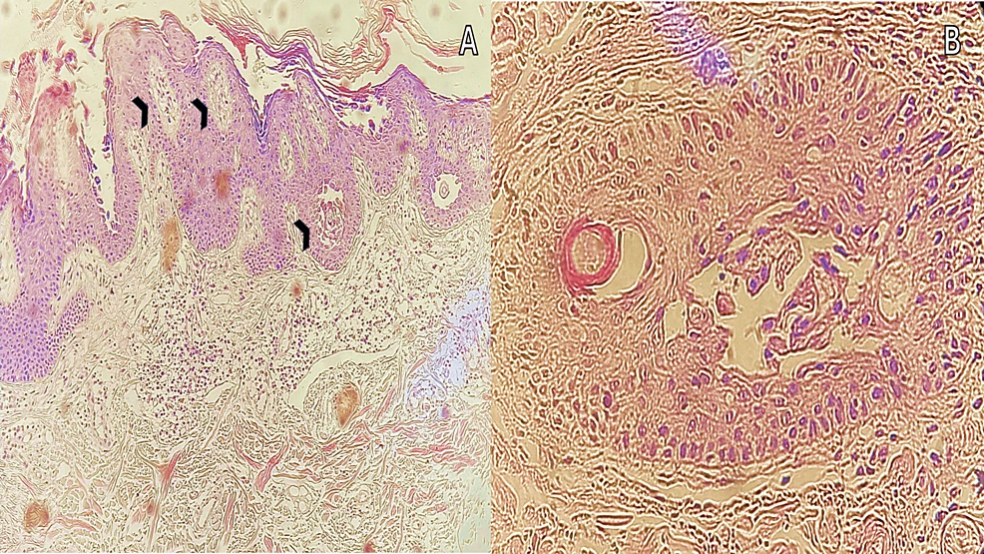
Histopathological examination showcasing intraepidermal blisters containing acantholytic and dyskeratotic cells; magnifications of 10 x (A) and 40 x (B) in hematoxylin and eosin stain

The biopsy revealed orthokeratosis, focal parakeratosis, intraepidermal blisters with acantholytic and dyskeratotic cells, and inflammatory mixed infiltrate in the dermis (Figure 2).

Paraclinical tests showed no abnormalities and tests for antinuclear antibodies and anti-double-stranded DNA were negative, as well as Gram staining. A probable diagnosis of seborrheic pemphigus was made, and direct immunofluorescence was requested for confirmation. Unfortunately, due to economic constraints, the patient reported being unable to undergo the test at the one-month follow-up. Nevertheless, notable improvement occurred with topical application of betamethasone valerate 1% cream twice daily.

## Discussion

In distinct subtropical areas of Brazil and Colombia, there's a relatively common endemic variant of PF, which leads to painful erosions referred to as "fogo selvagem" (Portuguese for wild fire) [1]. Conversely, pemphigus erythematosus (PE), also known as Senear-Usher syndrome) presents as a localized and sporadic form of PF, with lesions typically found on the face, upper trunk, and back [1].

Senear and Usher initially proposed in 1926 that PE presented a blend of pemphigus and cutaneous lupus erythematosus, characterized by a rash resembling lupus or seborrheic dermatitis [2,3]. However, this theory has since been discarded, as most PE cases lack serum antinuclear antibodies and fail to meet the American College of Rheumatology criteria for systemic lupus erythematosus [2].

Furthermore, since its initial description, numerous clinical reports have interchangeably used the terms 'pemphigus erythematosus,' 'seborrheic pemphigus,' or 'Senear-Usher syndrome.' This interchangeability may have resulted in some cases of seborrheic pemphigus being misclassified as PE or Senear-Usher syndrome. For instance, in 2014, Errichetti et al. reported only four cases of SP in the literature [4]. However, this data may be misleading, as there is no guarantee that these cases correspond to SP. Conversely, other cases of SP may have been erroneously labeled as PE or Senear-Usher syndrome. Furthermore, recent publications have persistently employed these terms interchangeably [5,6], even across diverse continents and countries [7].

Then, while the precise usage of SP terminology denotes a superficial and localized condition characterized by isolated erythematous and scaling lesions, primarily affecting the scalp, ear, and neck, histopathologically, serologically, and immunologically, it aligns with PF [2]. Additionally, in some other regions, distinctions have been appropriately made [8].

According to the 2013 validated European Guidelines on diagnosis and treatment of pemphigus, by the European Dermatology Forum in cooperation with the European Academy of Dermatology and Venereology, four criteria have to be considered to confirm the diagnosis, namely: (i) clinical presentation, (ii) histopathology (acantholysis at the granular layer), (iii) direct immunofluorescence (IF) microscopy, and (iv) serological detection of serum antibodies anti-Dsg1 by enzyme-linked immunosorbent assay (ELISA) or by indirect immunofluorescence microscopy [9]. 

In this case, unfortunately, we could not confirm the diagnosis. Thus, we would like to further discuss some other possibilities. Drug-triggered bullous eruptions are unlikely due to the absence of previous exposure by the patient. While pemphigus vulgaris typically affects the skin, it invariably involves the mucous membranes. However, in this patient, there was no mucosal involvement, a critical clinical distinction for accurate differentiation. Additionally, the presence of a pathognomonic acantholytic pattern, described as "tombstone-like," in the histopathology further supports discarding the diagnosis [2].

Bullous impetigo typically manifests as superficial vesicles and bullae, commonly localized around areas prone to skin breaks like the nose and mouth. Histopathological examination shows subcorneal or intraepidermal blisters with neutrophils and gram-positive cocci, indicative of bacterial infection. While microbiological culture is crucial for definitive differentiation in uncertain cases, in our situation, Gram stain and histopathological features were sufficient to discard the diagnosis [2].

Neutrophilic dermatoses should be addressed when detected by histopathology [2]. Cutaneous lupus erythematosus was one of the main differential diagnoses, given that the patient is a young woman; however, antinuclear antibodies and anti-double-stranded DNA were negative. Additionally, the histopathology presented an interface pattern, mucin deposits, and lymphocyte infiltration. Finally, there are other acantholytic dermatoses, such as Hailey-Hailey disease and Darier's disease, which present clues in the histopathological examination. In Darier's disease (keratosis follicularis), key features include acantholytic dyskeratosis with acantholysis accentuated in the lower epidermis, resulting in suprabasal clefting. Additionally, Darier's disease exhibits grains (basophilic keratinocytes with elongated nuclei in or near the granular layer) and corps rounds (dyskeratotic keratinocytes with a round nucleus surrounded by a blue rim or clear halo). On the other hand, Hailey-Hailey disease may demonstrate incomplete acantholysis, resulting in a lower epidermis appearance resembling a dilapidated brick wall. Although histopathological clues can provide valuable insights, it's primarily the clinical correlation with long-term evolution, dissemination patterns, and clinical history that distinguishes between these conditions, along with the absence of immunological and serological markers [10-12]. Notably, the absence of a known genetic background in the patient's family history is significant, except in the case of Grover's disease, which, like other acantholytic disorders, typically manifests as pruritic papules and papulovesicles primarily on the trunk of older white men. Involvement of other anatomical sites is infrequent, with only 24% affecting the face or scalp, and bullous presentation occurs in 8% of cases [11].

Like Grover's disease, Hailey-Hailey's disease, and Darier's disease, there are rare instances of involvement in seborrheic areas. There is no reported case of Darier's disease exclusively affecting the face [10], and only one case of Hailey-Hailey disease [12]. This underscores the importance of considering a range of factors, including clinical presentation and patient history, in making an accurate diagnosis.

Misunderstandings surrounding proper terminology emphasize the importance of distinguishing between the treatment approaches for SP and PE, given their distinct characteristics. While PE typically necessitates systemic therapy, our observations, along with others [13], suggest that SP can often be effectively managed with topical treatment alone. This is particularly due to its superficial and localized nature. Interestingly, this contradicts the European guidelines of 2013, which restrict the role of topical treatment to that of an adjuvant [9]. Nevertheless, the favorable responses observed in patients treated solely with high-potency topical steroids further validate this approach. However, further studies are needed to clarify if topical high-potency steroids are the best choice for SP. Nonetheless, accurately naming the terms for SD and PE is a crucial starting point.

## Conclusions

SP, a superficial and localized variant of PF, can often be effectively treated with topical corticosteroids alone and should not be confused with PE. Future research is needed to determine the best management strategies for SP, emphasizing the importance of accurate terminology in clinical practice and research.
